# A Conceptual Multi-Layer Framework for the Detection of Nighttime Pedestrian in Autonomous Vehicles Using Deep Reinforcement Learning

**DOI:** 10.3390/e25010135

**Published:** 2023-01-09

**Authors:** Muhammad Shoaib Farooq, Haris Khalid, Ansif Arooj, Tariq Umer, Aamer Bilal Asghar, Jawad Rasheed, Raed M. Shubair, Amani Yahyaoui

**Affiliations:** 1 Department of Computer Science, School of Systems and Technology, University of Management and Technology, Lahore 54000, Pakistan; 2 Department of Information Sciences, Division of Science and Technology, University of Education, Lahore 54000, Pakistan; 3 Department of Computer Science, Lahore Campus, COMSATS University Islamabad, Lahore 54000, Pakistan; 4 Department of Electrical and Computer Engineering, COMSATS University Islamabad, Lahore 54000, Pakistan; 5 Department of Software Engineering, Nisantasi University, Istanbul 34398, Turkey; 6 Department of Electrical and Computer Engineering, New York University (NYU), Abu Dhabi 129188, United Arab Emirates; 7 Department of Software Engineering, Istanbul Sabahattin Zaim University, Istanbul 34303, Turkey

**Keywords:** deep learning, advanced driving system, neural network, autonomous vehicle, intelligent driving system, reinforcement learning

## Abstract

The major challenge faced by autonomous vehicles today is driving through busy roads without getting into an accident, especially with a pedestrian. To avoid collision with pedestrians, the vehicle requires the ability to communicate with a pedestrian to understand their actions. The most challenging task in research on computer vision is to detect pedestrian activities, especially at nighttime. The Advanced Driver-Assistance Systems (ADAS) has been developed for driving and parking support for vehicles to visualize sense, send and receive information from the environment but it lacks to detect nighttime pedestrian actions. This article proposes a framework based on Deep Reinforcement Learning (DRL) using Scale Invariant Faster Region-based Convolutional Neural Networks (SIFRCNN) technologies to efficiently detect pedestrian operations through which the vehicle, as agents train themselves from the environment and are forced to maximize the reward. The SIFRCNN has reduced the running time of detecting pedestrian operations from road images by incorporating Region Proposal Network (RPN) computation. Furthermore, we have used Reinforcement Learning (RL) for optimizing the Q-values and training itself to maximize the reward after getting the state from the SIFRCNN. In addition, the latest incarnation of SIFRCNN achieves near-real-time object detection from road images. The proposed SIFRCNN has been tested on KAIST, City Person, and Caltech datasets. The experimental results show an average improvement of 2.3% miss rate of pedestrian detection at nighttime compared to the other CNN-based pedestrian detectors.

## 1. Introduction

Road safety in the modern world has been a challenging task that is required by all societies. Road accidents were noted to be the core cause of death in most urbanized and industrialized nations. According to World Health Organization (WHO), the total number of road accident casualties remains high, up to an unacceptable level of 1.2 million each year, and collisions with pedestrians take the 9th position among causes of death in the world [1]. Pedestrian detection based on computer vision technology has been the most researched subject over the last couple of years. One of the most significant reasons investigated in research studies is that pedestrian accidents occur due to unidentified pedestrian actions [2,3,4]. Pedestrian actions are based on environmental situations, circumstances, different activities, and variations such as poses, dressing styles, and occlusion [5,6]. The possible causes of distractions include eating, talking, using smartphones, and being under the influence of drugs or medication [7]. These distractions consequently engage pedestrian attention such as not paying full attention to their environment when they are passing through and crossing the road. These pedestrian behaviors create major demand for the detection of pedestrian actions for all Advance Driving Systems (ADS). The leading cause of accidents with pedestrians was the negative attitude of the pedestrian toward obeying traffic lights. In China [8], 60% of pedestrians have not followed traffic signal rules. Pedestrians feel comfortable especially when crossing roads in groups, which was related to the conformity phenomenon of psychology, creating an illusion of safety that encourages traffic violations.

### 1.1. ADS Challenges and Solutions

**Figure 1:**
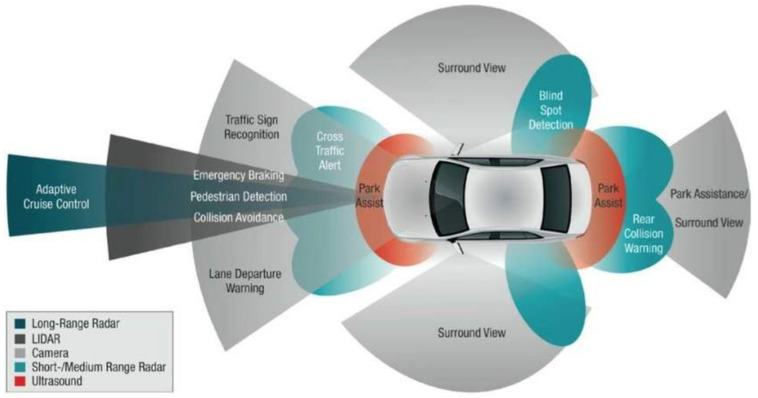
ADAS Sensing and driving assistance component [1].

**Figure 2:**
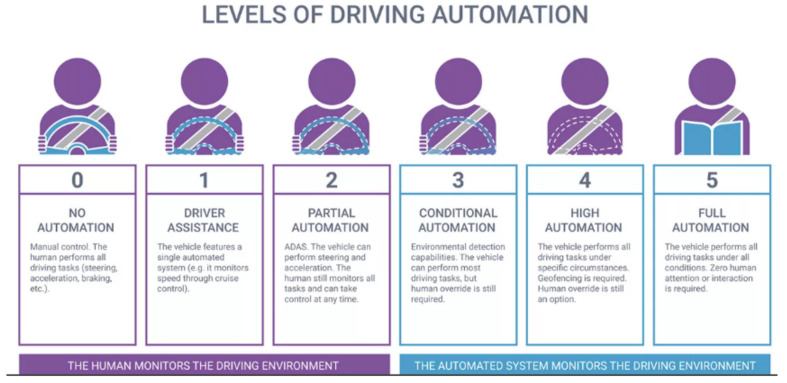
Levels of ADAS and Driving System [13].

To overcome pedestrian challenges ADS was used to avoid collisions with the pedestrian on the busiest road. The ADS vehicle recognizes the pedestrian’s actions to avoid a collision that causes accidents. The ADS utilizes multiple sensors, such as LiDAR, camera, and radar that facilitate the vehicle’s vision capacity as shown in Figure 1. Furthermore, the detected objects from captured images were processed instantly to enable the vehicles to make timely decisions [9,10,11]. In addition, autonomous vehicles were expected to deal with chaotic roads, full of pedestrians and dense traffic, which requires that captured must be processed in real-time. The objective of ADS, deployed in vehicles for the detection of pedestrian actions is to minimize accidents and enhance safety. The ADAS used in ADS for providing extra services such as sound alert systems, warnings, or vibrations that aids the driver in the event of a collision detected by Collision Mitigation Systems (CMBS) [7,9,10]. The level and components of ADAS help in detecting activities, operations, and overall judgment related to the environment as shown in Figure 2. Level 0 (no automation) demonstrates the services of the vehicle have been performed by a human. Level 1 (information presentation) demonstrates how ADAS offer the least possible driver assistance. At level 2 (avoidance of hazards), ADAS give helps drivers by assessing the dangers on the roads through alerts and also provides help in unusual threatening situations. Level 3 (Emergency braking control) shows how ADAS give additional assistance to drivers through different forms of control measures and mitigates or avoids the potential dangers actively with little or no significant driver input. Level 4 (High Automation) shows that the system will be requested by the driver to act under certain conditions. Level 5 (Full automation) demonstrates all the activities that have been performed by the vehicle in all conditions. The progress in ADAS technologies has been notably significant and contributes to the minimization of accidents as a result of vehicle coalition and general road carnage by about 40% [1]. The ADAS requires maintaining a huge software stack for successfully executing the right decisions in real-time like emergency braking [11,12].

### 1.2. Night Time Object Detection Challenges

The nighttime detection of pedestrians has been a challenging task in all conventional pedestrian detection algorithms. There are several examples, having different performance rate at morning and night time detection. For instance, the SDS R-CNN algorithm reported a 7.36% miss rate for the daytime dataset and increased 64% on nighttime detection [14]. Moreover, we can enlist the major challenges in autonomous and surveillance scenarios as:

- Viewpoint: while discussing night-time pedestrian detection, video frames are captured on small scale. Moreover, rain and occlusion are also some of the challenge cases due to poor visibility. Capturing downward and parallel angles in some cases also causes a higher miss rate in detection.
- Imbalance illumination: Light distribution is strenuous in different angles and also imbalance during nighttime because the main light source is the vehicle itself and street lights. These may cause false detection and poor detection rates.

In the past few years, deep learning has been used for the enhancement of nighttime pedestrian detection. The Region-based Fully Convolutional network (R-FCN) [2], Fast R-CNN [13], Mask R-CNN [15,16], Residual Neural Network (ResNet), and [17], LeNet [18], were state-of-the-art frameworks that have been used for the detection of nighttime pedestrian detection. LeCun et al. [9] suggested the LeNet architecture for the detection of nighttime pedestrian detection. It consists of only two convolutional layers, and it uses the tanh activation function that decides whether the input layer neuron has been activated or not. However, due to only two layers, LeNet was used for the small training dataset and has fewer processing capabilities. Jonathan et al. [11] proposed an R-FCN framework for the semantic segmentation from the images to detect pedestrians. The R-FCN architecture consists of two processes i.e., convolution and upsampling. The convolved images that are un-sampled from the convolutional layer have been fused to get a more accurate prediction. After the unsampled process, the output has been pixel-wise segmented images. However, this framework failed to detect the region before convolution because the huge region has been selected for the pedestrian in an image. Kaiming et al. [7,19] introduced the Mask R-CNN for the instance segmentation and detection of a moving and non-moving pedestrian in an image. The feature maps were identified by Region of Interest (ROI) pooling, and SVM has been used to extract the high-level feature for the classification of images. However, the localization of instance segmentation is not very easy in hazy appearance, blurred and unclear boundaries, overlapped pedestrian in-stances, and large/small instances due to their different characteristics. Ross Girshick et al. [18,20] proposed the Fast R-CNN architecture that has been used to solve the scale awareness problem with multi-scale filters on all the objects in an image. The convolved images have been generated with the help of CNN and the feature map of these images has been identified by ROI pooling. These feature maps are then fed into the fully connected layer that predicts the class of the object in an image. The results have been represented by bounding boxes, but this process has not been used to efficiently detect pedestrians of different sizes due to the heterogeneous size of instances that affects the performance of the network to detect the pedestrian in the image.

## 2. Scale Invariant Faster Region-Based Convolutional Neural Networks (SIFRCNN)

**Figure 3:**
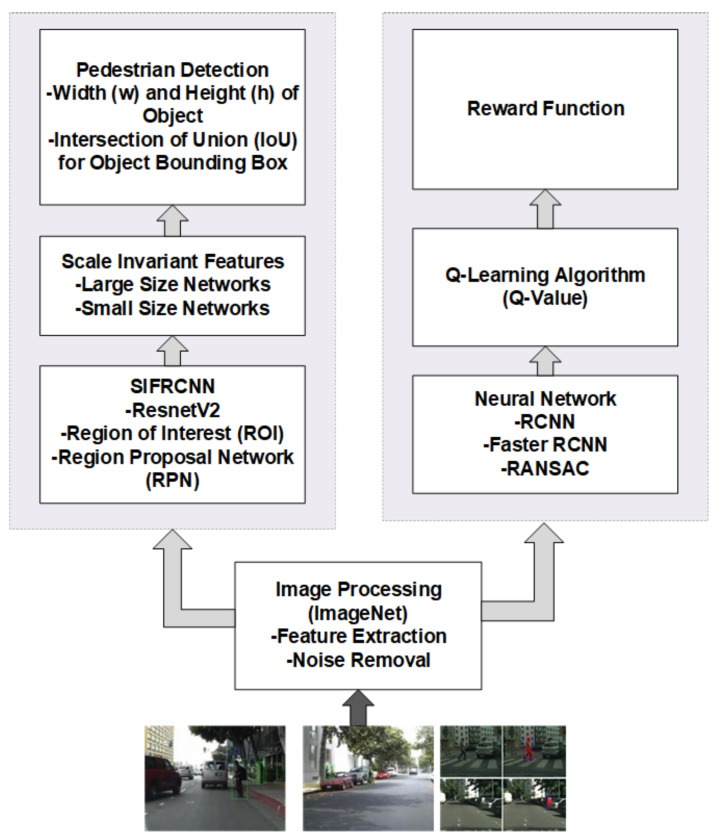
Flow diagram of complete SIFRCNN (Proposed) Algorithm.

The proposed framework SIFRCNN has been used to address the existing technical issues as described in Table 1. Initially, the term scale-invariant convolutional neural network (SiCNN) was introduced by [21]. But this model was based on CNN and was worked to process multi-scale feature exaction for appropriate decision making and classification. However, speed, accuracy, and robustness don’t have promising results due to the number of layers and multi-stage processing delays. Thus, we have proposed and combined to use the Scale Invariant (SI) parameters with Faster Region-based Convolutional Neural Networks (FRCNN), and named our proposed framework as Scale Invariant Faster Region-based Convolutional Neural Networks (SIFRCNN). Moreover, we also have added deep reinforcement learning (DRL) for reward functioning to perform better functions of autonomous vehicles including control, driving, and navigation as evince in Figure 3.

**Figure 4:**
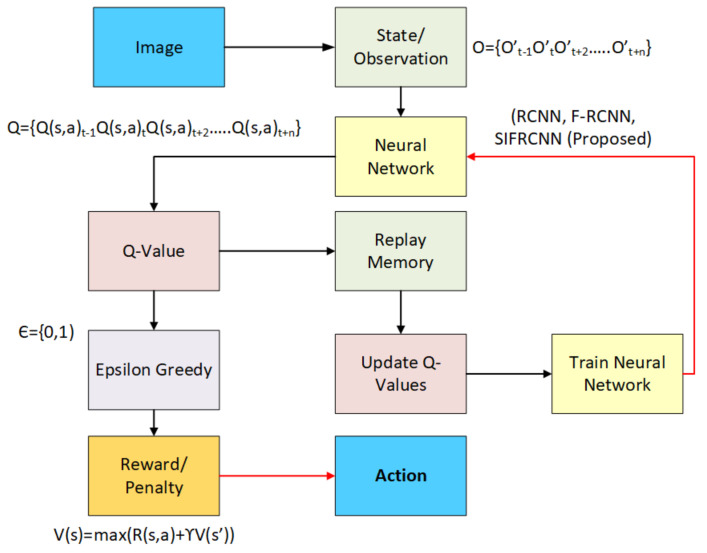
Flowchart of Reward Function by Proposed Method (SIFRCNN).

Moreover, the scale-invariant features with aster Region-based Convolutional Neural Networks (FRCNN) have added the following capabilities in previously available models and thus proved the novelty of the proposed architecture:

- ROI Pooling layer to extricate the equal-length vectors of image frames.
- Build a single-stage network to manage the segmentation and feature extraction neither like RCNN where it involves multiple stages, for instance, feature extraction, region generation, and classification.
- FRCNN has enabled to share of the computational load by using the ROI Pooling layer on the single network of proposals and has a much more accurate comparison to CNN.
- SIFRCNN has separated the action space into detection and operation as illustrated in Figure 3 and Deep Reinforcement Learning (DRL) to improve the accuracy and efficiency of operational features as defined in Figure 4.

The novelty of our work is that SIFRCNN was efficiently used to detect real-time pedestrian actions in different environments and types of pedestrians such as a child, an adult, and senior citizens, especially at nighttime. Furthermore, the RL has been used to effectively detect the state actions and rewards from the environment. The SIFRCNN indicates better accuracy in detecting objects in ADS as compared to other CNN-based detectors. The major focus of this article is to investigate critical issues of nighttime pedestrian detection of actions. We have proposed a framework to efficiently detect nighttime pedestrian actions at different scales. The paper has addressed the state-of-the-art technological developments incorporated in ADS to aid in sensing nighttime pedestrians on the road, capturing their images of different scales, observing, and more importantly, minimizing disaster occurrence to ease life. The SIFRCNN first passes the raw images through the feature extractor called image to extract the classes of the objects in the images. These classes pass through the convolution layer to extract their whole feature maps. This feature map is then passed over to RPN to generate the feature map of image visibility and scale invariant feature map to get the full-scale aware information of the pedestrian in an image. Furthermore, these images from the environment go to the vehicle agent as a state action and then the vehicle agent decides the best action to get the optimized reward as shown in Figure 4. The RL has been used in our proposed framework that is applied in an autonomous vehicle for trajectory optimization; path planning and motion planning are outstanding for multiplex navigation. The data from the environment and our proposed framework has been used for the prediction of nighttime pedestrian actions on the road. The positioning, heading, and velocity of the pedestrian towards the vehicles are the state spaces that have been used in ADS to get the rewards to enhance safety [22,23].

The major contribution of this study is as follows:

- This research presents the state-of-the-art SIFRCNN algorithm to detect real-time pedestrian action detection in different environments i.e., types of pedestrians such as a child, an adult, and a senior citizen, especially at nighttime. Moreover, SIFRCNN used two learning approaches to integrate the benefits into real-world solutions including autonomous navigation, hindrance detection, and avoidance in stochastic environments. The main target of the algorithm is to achieve ADS level 4 with high automation.
- Real-world training and validation of CityPerson, Caltech [21,24], and KAIST data-set have been simulated to check the efficiency of our proposed algorithm.

The rest of the paper is organized as follows: following the introduction section, we present the related work in Section 3. The proposed framework has been discussed in detail in the Section 4 “proposed framework and architecture” where we have a detailed explanation of pedestrian detection at nighttime. The datasets and experiments along with their properties have been presented in the Section 5 “Experiments and Results”. Finally, we present the conclusion and future directions of the research work in the Section 6.

## 3. Historical Notes and Related Work

The most recent research studies on the subject matter indicate that pedestrian detection based on deep learning was proposed for improving accuracy in pedestrian detection [11,13]. However, the pedestrian detector still suffers from various issues caused by the scales of a pedestrian in the image, pedestrian illuminating variation, pedestrian occlusion, and complex background in the image. These issues have been partially addressed and significantly affect the performance of ADS in pedestrian detection. Jianan Li et al. [12,20] proposed a scale-invariant feature generator that has been used to detect scale and multiple pedestrians respectively as compared to existing pedestrian detectors and instance segmentation approaches. However, these approaches have been used only for a wide range of input scales but are not suitable for short scales. Shaoqing Ren et al. [21,22] presented Faster RCNN to detect the nighttime pedestrian by using visible light cameras. This method fused the deep convolutional features of successive images from the feature extractor. This method is robust for all pedestrian detection in all lighting conditions. However, this method has not worked for the size of the pedestrian in the image. Developing convolutional neural networks (CNN) using the classification algorithm and the deep convolution feature, such as Support Vector Machines (SVM), has significantly contributed to pedestrian recognition and detection. However, this has not been used for nighttime pedestrian actions while it is more efficient in daytime pedestrian detection.

In addition, none of the vehicles were effective in detecting pedestrians crossing the road at night. According to Girshick et al. [2], the number of low-intensity pixels is higher in nighttime images, which results in low noise and contrast. One possible solution to reducing the noise is increasing camera exposure. Yet, doing so would increase motion blurring, making pedestrians hardly recognizable [4,25,26]. Maintaining the exposure level would also make it difficult to obtain important features. Krishtopik et al. [23] noted that the collected data contains true and false edges that arise from the image’s actual contents and noise, respectively. Current nighttime detection technologies and research have been categorized as either multiple-camera or single-camera methods. Multiple camera devices extract and fuse each image’s features using infrared and visible light technology, whereas single-camera methods use low-image resolution devices such as far infrared (FIR) cameras [24]. However, FIR-based cameras can only extract a few pedestrian features when employed in long-distance human detection. One of the challenges that the proposed system aims to solve is pedestrian detection at night. As demonstrated by existing studies and technologies of automation, the Near Infrared (NIR) illuminator’s functionality is limited in low external light. The device’s power and distance and angle of illumination are contributing factors to its limited functionality [11,27].

However, we have proposed a framework that overcomes this challenge by using a visible-light camera and SIFRCNN. The summary of technical issues in previous techniques and their solution by SIFRCNN has been presented in Table 1. The SIFRCNN is composed of a feature extractor, an (RPN), and a classifier. In the proposed system, the feature extractor will generate feature maps by receiving multiple pedestrian images and repeatedly passing them through the convolution layers, Rectified Linear Unit (ReLU), and the max pooling layer. Successive feature frames from the extractor are fused and used as input by the RPN and classifier. The novelty of this work is that our proposed framework strongly incorporates nighttime pedestrian detection. This effectively helps to detect the nighttime pedestrian actions, as well as used to find the height of the pedestrian in the image through scale invariant. The next section describes the proposed SIFRCNN framework briefly.

## 4. Proposed Framework and Architecture

**Figure 5:**
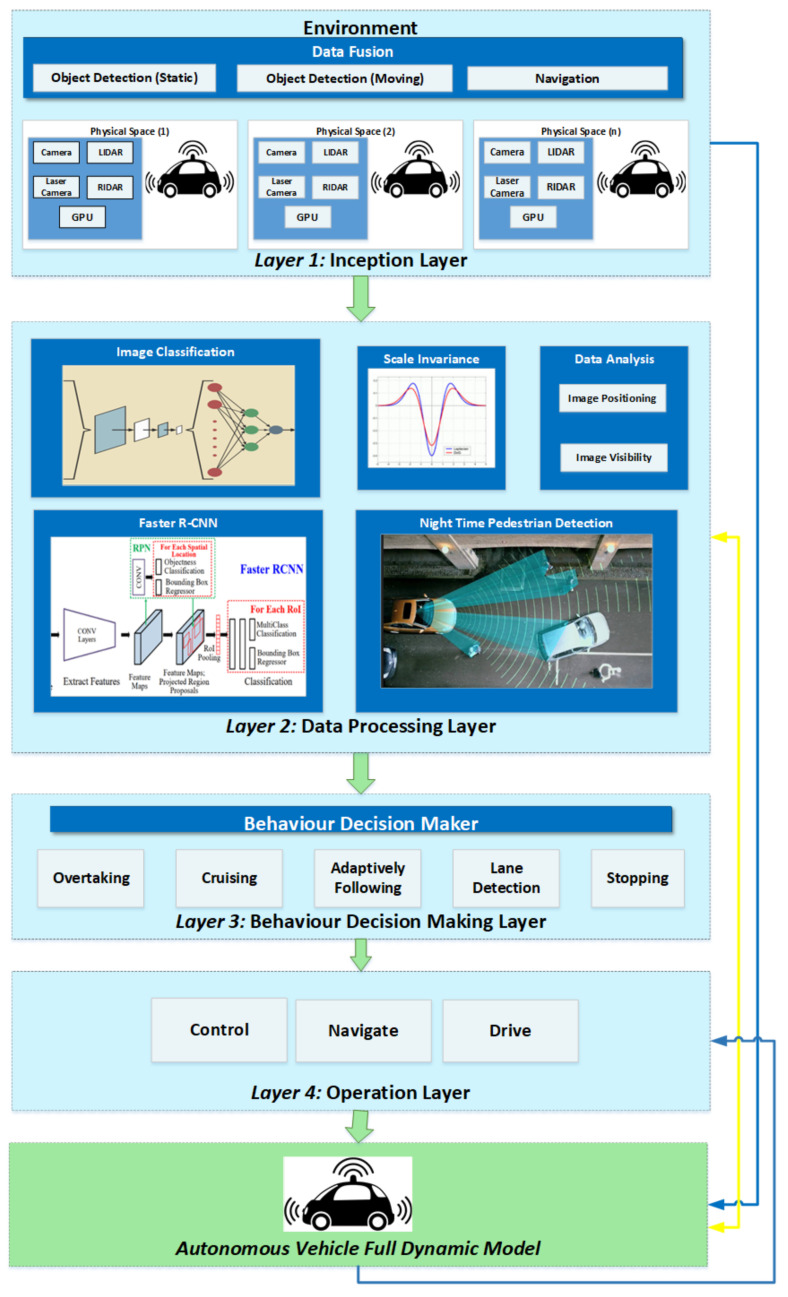
Proposed layer diagram for an autonomous vehicle by using deep reinforcement learning.

In this section, we present the proposed framework to detect nighttime pedestrian actions on the road. Furthermore, the technique used for the detection of pedestrian actions has also been presented. Lastly, we analyze all the results and assign them to the vehicle as an agent to train themselves and get the maximum rewards. Our proposed framework comprises three main sections the faster R-CNN, scale-invariant feature zone, and reinforcement learning. The Faster R-CNN extracts the feature from the images and then makes the spatial and temporal feature map, whereas the scale-invariant extract feature by assigning the weighted value to estimate the size of the pedestrian and then the results as a state of action accept the vehicle as an agent to train itself from the environment. In our proposed framework we have defined four layers that describe the detailed processing for the detection of pedestrian actions as illustrated in Figure 5.

### 4.1. Inception Layer

The inception layer is the fundamental layer to enable autonomous vehicles that tells us about the driving environment, location, surrounding obstacles, and prediction of the coming state. The inception layer of the proposed architecture gets raw images from the environment and after processing presents fused images to the data processing layer that finds the actual image action in the night view. This layer uses ADAS sensing technologies such as cameras, laser radars, and as well as millimeter-wave radars to assist in enhancing safe driving. These ADAS components are embedded in the proposed framework for accurate measurement of the distances between the autonomous vehicle and pedestrians. The information has been obtained from the environment via vehicle sensors such as LIDAR, radar, cameras, and GPS. The short/long radar and ultrasonic sensors have been used to detect the action of the pedestrian on the road as shown in the Figure 5 inception layer. Visible light cameras collect the images by using 400–700 nm wavelength and convert them into signals to process and have the capability to capture static images and dynamic images such as videos as well. Also, have better visibility and object target capability for better surveillance. Thus, for these properties in this study, we have used the visible light camera to capture the static and dynamic behavior of the object.

### 4.2. Data Processing Layer

**Figure 6:**
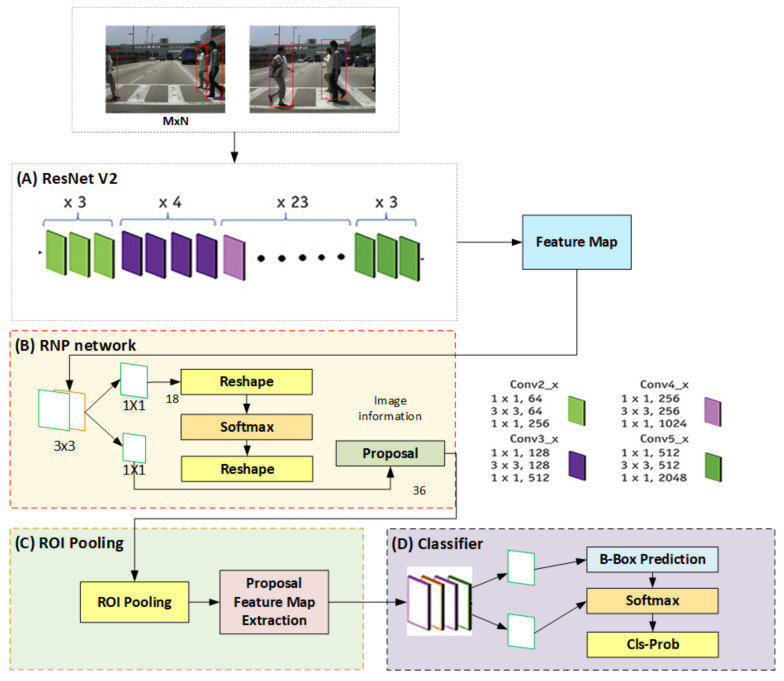
Complete Faster R CNN architecture.

The second layer of the proposed framework presents the different processing applied to the fused images from the inception layer. The data processing layer first, analyzes the fused image in which their positioning and visibility in the fused image. secondly, after analysis of the image positioning and visibility then sends it to the image processing phase where the image classifier extracts the high-order features class and then forwards it to the SIFRCNN where the Resnet v2 is used to extract the feature and then forward to the ROI pooling where it process to produces anchors of different sizes and produces the temporal and spatial feature map as shown in data processing layer Figure 3 and Figure 6, In the end, with the help of the scale-invariant feature zone assigned the weighted values to recognize the heights of the pedestrian.

#### 4.2.1. Image Processing

The major methodology for data processing in SIFRCNN can be used for image classification with the help of imageNet. This helps the autonomous vehicle to increase its accuracy to detect the object’s actions in the image. The imageNet provides an efficient classification of road objects that involves pedestrian actions from complex scenes and scenarios on road. The Faster R-CNN process is the second consideration of the SIFRCNN framework that involves two stages: (RPN) and (ROI) polling layer that processes proposals process with the help of imageNet with digresser and final classifications. Moreover, SIFT (Scale Invariant and Feature Transform) and HOG (Histogram of Oriented Gradients) are well-known methods for object detection. SIFT uses 16*16 frames and is divided into 16 cells whereas HOG uses the features of the complete image and divides into smaller blocks as cells and processes the gradients for each pixel by summing us for each cell in a pixel. However, in this study, we are using the IoU scale-invariant feature to extract the features of an object [28]. Moreover, The two-stage Faster R-CNN processes function on feature maps retrieved from an image classifier preserved for classification activities, as shown in Figure 5 data processing layer. The Inception Resnet v2 is used as a component feature extractor because of its ability to attain high levels of accuracy in classification activity. Each location in the Faster R-CNN produces a defined number of anchors corresponding to various scales and elementary aspect ratios from the feature map as shown in Figure 5. These were able to provide bounding boxes of various sizes and ratios. These boxes were used to capture the scale and aspect ratio of the pedestrian actions in the image. The optimized performance of the object detection anchor generates fewer anchors effectively for detecting clear foreground objects from images. Four basic parameters (w,h,x,y) have been used in the anchor method, where w and h are the height and width of the anchor whereas *x* and *y* are the axes of the anchor. Equation (1) shows the simple formula to generate an anchor.

(1) $$\frac{S(w,h,x,y)}{I}=\frac{S(x,y)}{I}\times \frac{S(w,h)}{x,y,I}$$

The Intersection of Union (IoU) is a method used to calculate which object bounding box for each anchor box has the highest overlap divided by non-overlap [10]. This method cannot be used directly to find the value of *w* and *h* because in ground reality the value of *w* and *h* are unknown for the detection of pedestrian action. The IOU between the variable anchor and the ground truth bounding box, which is denoted by vIOU, is used to find the value of w and has shown in Equations (2) and (3):

(2) $$vIoU\left(b_{wh},gt\right)=maxIoU_{normal}\left(a_{wh},gt\right)$$

(3) $$L_{shape}=L1(1-min\left(\frac{w}{w_{g}}\right),\left(\frac{w_{g}}{w})\right)+L1(1-min\left(\frac{h}{h_{g}}\right),\left(\frac{h_{g}}{h})\right)=L1\left(1-min\right)$$

In our proposed framework, nighttime pedestrian detection functionality has been based on the IHS transform-based image fusion of successive image features. The functionality of nighttime pedestrian detection can be summarized in four stages: (i) spatial prepossessing and feature extraction, (ii) temporal fusion of feature maps, (iii) RPN and classification, and (iv) final detection of pedestrians. These stages are based on feature fusion in nighttime pedestrian detection. Firstly, we propose the fusion of successive image frames to improve object detection. It converts the single nighttime image into frames to enhance the brightness and contrast values to detect pedestrian action. Then the features of successive images can be combined by using weighted summation to accurately produce bounding boxes during the movement of the camera and the pedestrian. The depths of the RPN and classification were directly proportional to the receptive field of image scalar features. The scalar features of the same position of a pedestrian in the environment were likely to have similar contents in the receptive fields of an image. However, the use of CNN in the proposed framework provides robustness against translation and transformation of nighttime pedestrian actions, which increases the features fusion and reduces noise.

**Figure 7:**
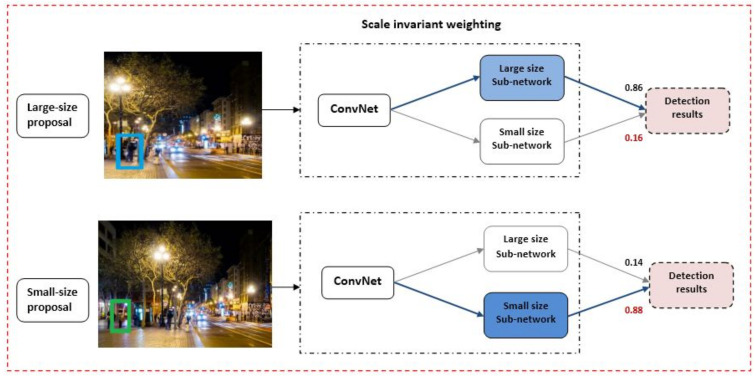
Scale-invariant feature of the proposed model.

The scale invariance feature consists of large and small-size networks that detect height differences in nighttime pedestrians as shown in Figure 7. The height of the pedestrian in the image has been obtained by combining the outputs of the two large and small-size networks of height variation and the object size is measured by its width and height. The height of the bounding box further varies with the distance between the pedestrian and the camera while the width is determined by the pedestrian’s action.

The input features have been extracted sequentially, first through the convolutional layers and second by the maximum pooling layers. The extracted features were fed into the scale weighting networks for the classification of scale invariants. The convolution layers were further utilized in each sub-network to extract scaled features. An RoI pooling layer then pooled the resulting feature maps into a fixed-length vector. It concludes that two fully connected layers were also used to perform scale-aware detection; given k object classes, the first network gave the output classification results while the second network produced bounding boxes for each pedestrian action. Finally, each output from these networks was weighed to obtain the detecting results as shown in Figure 6.

#### 4.2.2. Data Analysis

Data analysis is based on real-time processing to detect the pedestrian action from the SIFRCNN as shown in Figure 3 data processing layer. In this process, the positioning and visibility of the pedestrian in an image have been analyzed. Furthermore, visibility and positioning information is also sent to the behavioral decision-making layer which decides the behavior of autonomous vehicles based on nighttime pedestrian actions.

### 4.3. Behavior Decision Maker Layer

The behavior of the vehicle was determined by a data analysis module based on information from the surrounding environment and pedestrian actions. Behavior decision-making layers have four states for the vehicle: (i) overtaking, (ii) lane detection, (iii) adaptive following, (iv) stopping. The overtaking maneuver was triggered when the pedestrian was detected from the environment. Lane change action occurs when the distance between the pedestrian is greater than the adjacent lane only if the adjacent lane was available otherwise vehicle stopped. Lane detection is the core functionality of the autonomous vehicle while moving on the road. The vehicle has crossed the originating pedestrian and maintained a safe distance gap and then after crossing, the vehicle moves back to the original track. The Adaptive Following process handles the movement of the vehicle if one or more pedestrians have been detected moving toward the vehicle in the same lane. The vehicle follows the action of the pedestrian and decides either to slow down the speed or change the lane.

### 4.4. Operation Layer

The Operation layer is the last in our proposed framework that performs against the pedestrian action to avoid accidents on the road. This layer decides which operation should be adopted based on the environment and the data from the SIFRCNN. Control, navigation, and driving were considered the basic operations of the vehicle. This layered work with the sophisticated algorithms of SIFRCNN to perform these features with optimal accuracy. The decision of immediate operation that was triggered when the gap between the pedestrian is less than the safe lane-change distance is totally dependent on the results and detection of predecessor layers. This decision plan may lead to a minimal accidental situation.

### 4.5. Data Evaluation Algorithm

We have used two data evaluation algorithms (i) Random Sample Consensus (RANSAC) algorithm and (ii) the Q-learning algorithm. The RANSAC algorithm has been used to produce maximum inlier to identify a plane whereas the Q-learning algorithm has been used to find the state and actions of the vehicle agent to get maximum rewards.

#### Random Sample Consensus (RANSAC) Algorithm

The Random Sample Consensus (RANSAC) algorithm has been applied in the proposed framework to detect the randomly distributed object outliers. This approach utilizes an initial guess to provide a randomized sequence to enhance nighttime pedestrian detection approximations. The RANSAC has been mostly applied in image processing to remove noise from a given dataset. The RANSAC algorithm has been used to select a hypothetical inlier subset. These image inlier subsets have been used to evaluate the model parameter. In this context, all the points within the estimated model have been classified as consensus sets as data inliers. The RANSAC process was applied to the input point sets to get maximum inliers to identify a plane as shown in Algorithm 1 (line 4—code listing 1). These inliers are then checked to form a set of inlier patches as shown in Algorithm 1 (line 7—code listing 1). The total number of patches has been determined in Algorithm 1 (line 9—code listing 1). The inner loop (13–21) shows the iterative clustering to find the inlier patches plane and this loop is repeated until the inlier patches were exhausted. The outer loop terminates when no significant patches were found.


**Algorithm 1** Pseudo code for RANSAC Algorithm (Code Listing 1: RANSAC algorithm)1 **Input:** point set A = {ai | i = 1,...,p}2 **Output:** planes Pj = {pjk |k = 1,...,Q}: for j = 1,...,J3 j = 1;4 **while** (!area(B) < A){5            m = 1;6            B = RANSAC Plane_Fitiing (A);7            C = Normal_Coherence_Check (B);8            D = Find_Largest-Patch (C);9            P = Patch_Number (C);10 **if** (area(D) = ?){11            A = A − C;12 **else**{13 **while** (area(D) > ? and m < M){14            F = Iterative_Plane_Clustering (A,D);15            Pj = Find_Largest_Patch (F);16            A = A − Pj; C = C − D;17            D = Find _Largest_Patch (C);18            j = j + 1 ; m = m + 1;19 }20 }21 }


### 4.6. Reinforcement Learning for Autonomous Vehicles

The RL is a machine learning paradigm that analyzes the actions of vehicle agents that maximize the notion of cumulative reward. In the learning system, the behavior of the vehicle was influenced by its later inputs and the learner’s actions. The vehicle must identify the most appropriate action through the trial-and-error method. The vehicle perceived its environment through sensors and our proposed framework performs actions within the environment through actuators. The vehicle must perform tried actions that were discovered to be effective in producing rewards. The vehicle not only exploited past actions to get rewards but is also keen to explore better future actions. RL has been beneficial in overcoming some of the challenges that were presented by the proposed system. For instance, a vehicle agent learned the best action to avoid accidents by exploiting its pedestrian encounters. Table 2 shows various actions that were performed by an autonomous vehicle using DRL. These actions encompass state spaces, action spaces, motion planning, trajectory optimization, and rewards. Motion planning ensures that a trajectory exists between the target point and destination while state spaces enable the vehicle to change its lane. These actions were used to facilitate smooth operations of the vehicle when combined with state actions and motion planning.

#### Q-Learning Algorithm

The Q-Learning algorithm has been applied in the proposed framework to get an optimized reward from states and actions. The RL agents may learn policies and values function directly from the environment. The temporal difference (TD) method enables the RL agent to predict the expected values from the sequence of states to work in non-episodic conditions [2,15,17,29,30]. The TD method is directly learned from raw experience without previous knowledge of the environmental state. The TD-based method is used in Q-Learning, which is a free value-based algorithm model that learns useful estimates from individual state-action pairs from the vehicle environment as shown in Equation (4). Q-learning learns the optimum state-action of vehicle values due to enough samples obtained from each state-action pair. The Q-learning algorithm is actively used for training and validating reinforcement learning agents. The state and action can be found in Algorithm 2 (code listing 2) with the help of policies derived from Q. The actions were applied by the vehicle according to the states and policies as shown in Algorithm 3, code listing 3. The acceleration and brakes actions were performed based on the velocity of the vehicle as shown in Algorithm 3 (line 7–12—code listing 3. These actions were then responded to with the angle named ɸthreshold to find the turning action as shown in Algorithm 3 (line 13–19—code listing 3). Algorithm 4 (code listing 4) shows the reward function for the vehicle agent. This implies the rewards according to their actions as shown in Algorithm 4 (lines 10–31).

(4) $$Q\left(s,a\right)\leftarrow Q\left(s,a\right)+\alpha \left[r+\gamma maxQ\left(s^{\prime },a^{\prime }\right)\right]-Q\left(s,a\right)$$

**Algorithm 2** Pseudo code for learning algorithm for the selection of state action, policies, and rewards (Code Listing 2: Deep Q-learning algorithm)1 Initialize P (state, action) with some random value2 **while** (P! = terminal){3     Initialize state4 **While** (state! = terminal){5     Choose an action from the state by policy inferred from P6     Take action to action, observer, state’7     P (state, action) ← P (state, action) + α [r + γ maxα,       P (state’, action’) − P (state, action)]8     state ← state’9  }10 }
**Algorithm 3** Pseudo code for Vehicle controller Algorithm (Code Listing 3: Hand Crafted vehicle controller)1 vehicle-controller-policy (st){2 vbrake ← 203 vmax ← 184 ɸthreshold ← 0.055 acc ← Do nothing6 steer ← Do nothing7 **if** (vt > brake){8     acc ← Brake9 **else if** (vt < Vmax){10    acc ← Accelerate11 **else if** (ɸt > ɸthreshold){12    steer ← TurnLeft}13 **else if** (ɸt < −ɸthreshold){14    steer ← TurnRight15    return (acc, steer)16   }17   }18 }
**Algorithm 4** Reward function for the vehicle agent (Code Listing 4: Reward Function)1 REWARD (st, at){2 vmin ← 53 vbrake ← 204 vmax ← 185 ɸthreshold ← 0.056 reward ← 07 **if** (vt > vbrake and acct = Brake){8    reward ← reward + 1}9 **else if** (vt > vbrake and acct = accelerate){10    reward  ← reward − 1}11 **else if** (vt > vmax and acct = Brake){12    reward ← reward − 1}13 **else if** (vt > vmax and acct = Accelerate){14    reward ← reward + 1}15 **else if** (ɸt < −ɸthreshold and steer t = TurnRight){16     reward ← reward + 1}17 **else if** (ɸt < −ɸthreshold and steer t = TurnLeft){18    reward ← reward − 1}19 **else if** (ɸt > −ɸthreshold and steer t = TurnLeft){20    reward ← reward + 1}21 **else if** (ɸt > ɸthreshold and steer t = TurnRight){22    reward ← reward − 1}23 **else if** (vt < vmin and acct ≠ Accelerate){24    reward ← reward − 125 return REWARD26 }


## 5. Experiments and Results

In this section, we present the comparison of the experiments on the pedestrian dataset for object detection and performance evaluation. The proposed system is trained on three datasets including, CityPersons [2,27,28,29], Caltech [21,22], and KAIST [31] and bench-marked against existing state-of-art R-CNN-based pedestrian detectors: R-CNN [28], Faster R-CNN [9,21,29,32], Fast R-CNN-M [33], and RPN+BF [34] and presented in Table 3 and Table 4.

### 5.1. Datasets

**Figure 8:**
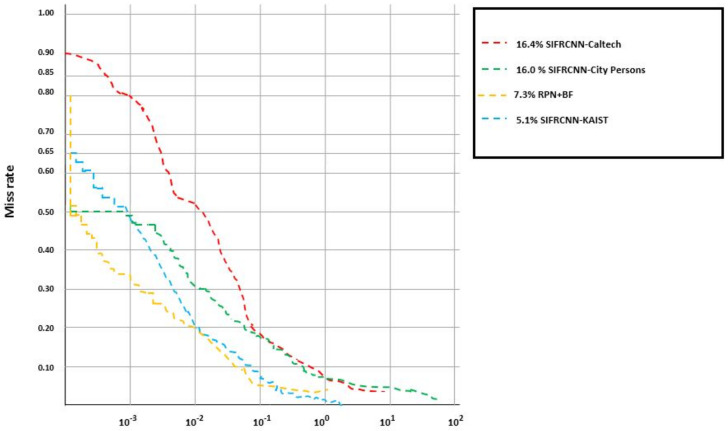
Comparison of state-of-the-art results on the KAIST test set at IoU = 0.50.

**Figure 9:**
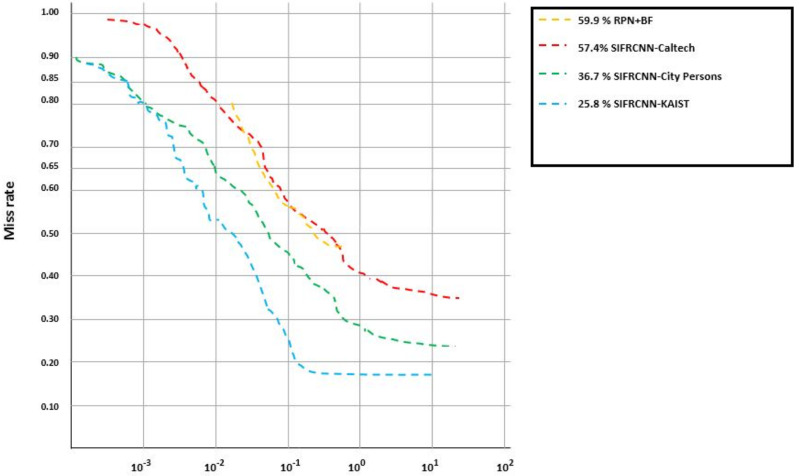
KAIST test set results at 0.75 IoU.

**Figure 10:**
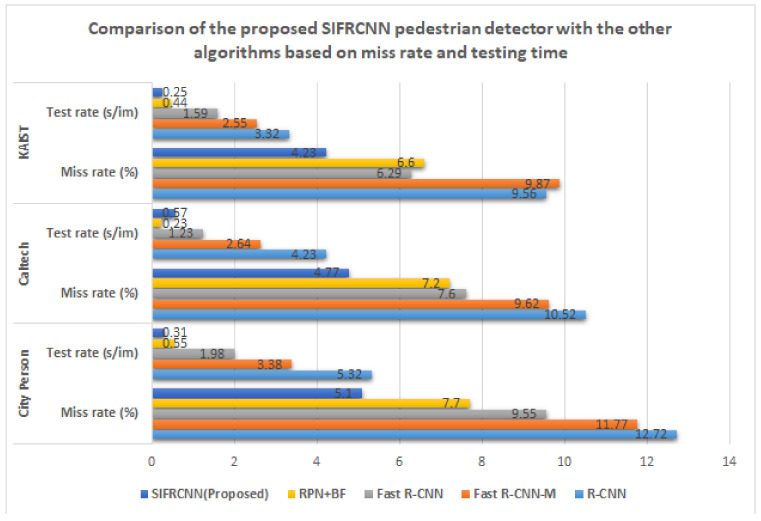
Comparison of the proposed SIFRCNN pedestrian detector with the other algorithms based on miss rate and testing time.

CityPersons: The CityPersons dataset was created for semantic segmentation in an urban area and consists of person annotations in a diverse set of video sequences. The videos were recorded in 27 different cities in Germany and neighboring countries. The dataset has 30 visual classes, each with 5000 images. The annotations are included in 20,000 images that have semantic labels CityPersons [2,28,29]. This performed additional training with City-Persons on nighttime pedestrian detection and obtains improved results for heavy occlusion, small scale, and higher localization quality cases (see Figure 8 and Figure 9). for this study, we only have considered the 17:00 to 05:00 next day time image frames. Specifically, using City-Persons for pre-training, the best model obtained a 16.0% log miss-rate (MRN) at an IoU evaluation of 0.50 and obtain a 36.7% long miss-rate at an IoU evaluation of 0.75 and presented in Figure 10.

**Figure 11:**
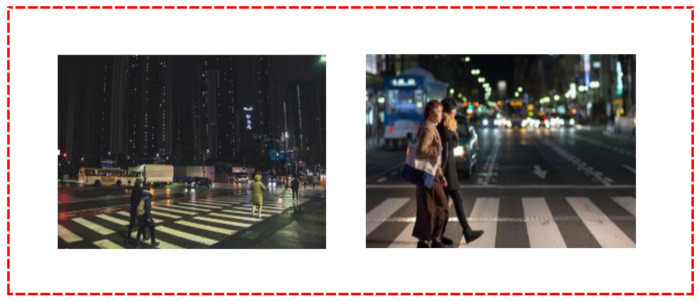
Nighttime image in the Caltech dataset.

Caltech: The Caltech pedestrian dataset contains close to 10 h of 640 × 480, 30 Hz videos that were captured by a vehicle driving through traffic in an urban environment [20,21,22,27]. A total of 350,000 and 2300 unique pedestrians have been annotated in about 250,000 frames—approximately 137-min-long segments—which implies that the dataset is useful in fusing the feature of successive images. The reference [16] used the dataset to train their model using nighttime images. The fusion of successive images model has been proved beneficial in training the model to detect pedestrians at night as shown in Figure 11. Moreover, for this study, we only have considered the 17:00 to 05:00 next-day time image frames.

KAIST: The KAIST dataset is used to perform nighttime pedestrian detection KAIST [14,30,35]. The KAIST dataset contains complex multispectral urban nighttime pedestrian action detection benchmarks. The dataset consists of 95,000, 640 × 480, 20 Hz, thermal pairs of videos that were taken during the movement of vehicles [4,13,29,36]. All the pairs were manually annotated for 103,128 dense annotations and 1182 unique pedestrian actions at nighttime.

### 5.2. Training Details

Hardware Specifications and Simulator: For the proposed model, a game engine-based simulator has been used and implemented by Unity ML agents o deal with the static environmental features. Whereas the GTA V game environment has also been used for dynamic model features. A summary of hardware specifications is presented in Table 5.

Data augmentation: Random cropping, flipping, scaling, and color space transformation have been applied for the images to reduce the cost and effort of training samples.

Anchor Designing: Following the [6,7], the anchor design strategy has been adapted for the pedestrian datasets. The complete designing process consists of three stages: (1) *Extraction of key frame*: enable the FFmpeg extraction from nine key frames per second from the selected range of image frames. (2) *Annotating bounding boxes*: For evaluation of all pedestrian objects at once, we have limited the multi-scale anchor scales from fine to coarse as 32 and $32\sqrt{2}$, 64 and $64\sqrt{2}$, 128 and $128\sqrt{2}$ respectively. This multi-scaling has eliminated image scanning by sliding window that computes the positions at separate scales and yet has better detection [37]. (3) *Manual Rectification*: A co-labeler tool has been used to ensure the correction of anchor boxes.

Loss Function: Occlusion is one of the key challenges for object detection including the inter and intra-class occlusion [14,15,38,39]. A loss function will be required to reduce the influence between objects. IoU scale-invariant feature has been used to effectively diminish pedestrian occlusion [12,39,40]. Two different scales IoU evaluation of 0.50 and 0.75 were applied to check the miss rate ratio and results are presented in Table 3. Moreover, Table 6 shows the comparison between the city person, KAIST, and Caltech datasets concerning the time of image capturing, the number of images, and the number of nighttime pedestrians that have been detected from the image. The KAIST dataset has been used for pre-training. The proposed SIFRCNN obtained a 5.1% log miss rate (MRN) at an IoU evaluation of 0.50 and obtain a 25.8% long miss rate at an IoU evaluation of 0.75.

## 6. Conclusions and Future Work

In this paper, we have proposed a framework for the detection of nighttime pedestrian actions for autonomous vehicles on the road to avoid accidents. This paper has investigated the possible improvements and enhancements of nighttime pedestrian action detection in ADS. To enhance the accuracy of detection and inference in challenging driving conditions such as relatively large-scale automobile variation, bad lighting conditions, and vehicle occlusion. We have named our framework SIFRCNN. SIFRCNN indicates better outcomes in both processing time and accuracy. The ROI Pooling functions have been used for the extraction of appropriately defined feature map and as well as utilizes the crucial features for accurate prediction of the end class label and the corresponding bounding boxes. The proposed framework is evaluated based on Caltech, City Person, and KAIST datasets. The experiment results show that the proposed network was simple, fast, and efficient. The incorporation of image capturing, sensing, and observation into the future ADS minimizes disasters and hence simplifies and saves a life. The future direction of this work is to introduce a model which will detect nighttime pedestrians in foggy, rainy, or snowy weather.

## Figures and Tables

**Table 1:** Technical issues in previous methods and their proposed solution by SIFRCNN.

Technical Issues	Proposed Solution	Implemented in SIFRCNN by
Camera related Problems	Heterogeneous Feature Extraction	IoU, Restnet V2
Camera Motion issues	Image stabilization	IoU, ImageNet
Object Deformations	Visual Segmentation	Scale Invariance Feature
Abrupt Motion detection	Intelligent detection Method	ROI, RPN
Background Scaling	Dynamic modeling Method	IoU, RPN, RCNN, Faster RCNN

**Table 2:** Automated tasks and their utilization in DRL.

Automated Task	Description
Motion planning	The vehicle learning agent learned to plan the trajectory and minimize the cost function to obtain smooth control behavior.
Overtaking	DRL has been used to learn an overtaking policy while avoiding accidents with pedestrians.
Lane keep	=RL system ensures that the vehicle follows the lane. Continuous action provided a smoother path to learning, while more limited conditions were slow the vehicle agent learning.
Lane change	When changing the lane or accelerating, Q-learning is used to learn which path to be followed.

**Table 3:** Comparison of the proposed SIFRCNN pedestrian detector with the other algorithms based on miss rate and testing time.

Dataset	Method	R-CNN	Fast RCNN-M	Fast R-CNN	RPN+BF	SIFRCNN (Proposed)
City Person	Miss rate (%)	12.72	11.77	9.55	7.7	5.1
	Test rate (s/im)	5.32	3.38	1.98	0.55	0.31
Caltech	Miss rate (%)	10.52	9.62	7.60	7.2	4.77
	Test rate (s/im)	4.23	2.64	1.23	0.23	0.57
KAIST	Miss rate (%)	9.56	9.87	6.29	6.6	4.23
	Test rate (s/im)	3.32	2.55	1.59	0.44	0.25

**Table 4:** Comparison of results by applying SIFRCNN (Proposed Algorithm), RCNN, Faster RCNN-M, Faster RCNN, RPN+BF on CityPerson, Caltech, and KAIST datasets.

Dataset	Method	Anchor Scale	Small (mAP%)	Large (mAP%)	IoU
City Person	RCNN	128${}^{2}$ 256${}^{2}$ 512${}^{2}$	10.11%	35.12%	0.3
		64${}^{2}$ 128${}^{2}$ 256${}^{2}$	16.11%	32.57%	0.44
		16${}^{2}$ 64${}^{2}$ 128${}^{2}$	15.80%	31.63%	0.68
	Faster RCNN-M	128${}^{2}$ 256${}^{2}$ 512${}^{2}$	12.56%	29.66%	0.23
		64${}^{2}$ 128${}^{2}$ 256${}^{2}$	16.33%	28.32%	0.56
		16${}^{2}$ 64${}^{2}$ 128${}^{2}$	12.99%	33.80%	0.3
	Faster RCNN	128${}^{2}$ 256${}^{2}$ 512${}^{2}$	19.23%	22.23%	0.5
		64${}^{2}$ 128${}^{2}$ 256${}^{2}$	11.25%	30.33%	0.53
		16${}^{2}$ 64${}^{2}$ 128${}^{2}$	11.02%	20.33%	0.63
	RPN+BF	128${}^{2}$ 256${}^{2}$ 512${}^{2}$	10.23%	33.20%	0.31
		64${}^{2}$ 128${}^{2}$ 256${}^{2}$	18.66%	38.22%	0.44
		16${}^{2}$ 64${}^{2}$ 128${}^{2}$	18.23%	35.00%	0.5
	SIFRCNN (Proposed)	128${}^{2}$ 256${}^{2}$ 512${}^{2}$	35.44%	59.63%	0.79
		64${}^{2}$ 128${}^{2}$ 256${}^{2}$	60.55%	60.59%	0.8
		16${}^{2}$ 64${}^{2}$ 128${}^{2}$	66.35%	62.56%	0.76
Caltech	RCNN	128${}^{2}$ 256${}^{2}$ 512${}^{2}$	25.00%	38.30%	0.5
		64${}^{2}$ 128${}^{2}$ 256${}^{2}$	28.20%	40.55%	0.2
		16${}^{2}$ 64${}^{2}$ 128${}^{2}$	18.50%	33.65%	0.37
	Faster RCNN-M	128${}^{2}$ 256${}^{2}$ 512${}^{2}$	10.22%	34.50%	0.4
		64${}^{2}$ 128${}^{2}$ 256${}^{2}$	29.78%	35.90%	0.3
		16${}^{2}$ 64${}^{2}$ 128${}^{2}$	19.36%	44.20%	0.25
	Faster RCNN	128${}^{2}$ 256${}^{2}$ 512${}^{2}$	18.22%	39.50%	0.22
		64${}^{2}$ 128${}^{2}$ 256${}^{2}$	13.05%	29.60%	0.44
		16${}^{2}$ 64${}^{2}$ 128${}^{2}$	18.23%	41.02%	0.38
	RPN+BF	128${}^{2}$ 256${}^{2}$ 512${}^{2}$	13.08%	35.02%	0.5
		64${}^{2}$ 128${}^{2}$ 256${}^{2}$	29.88%	28.80%	0.49
		16${}^{2}$ 64${}^{2}$ 128${}^{2}$	18.50%	25.60%	0.35
	SIFRCNN (Proposed)	128${}^{2}$ 256${}^{2}$ 512${}^{2}$	39.20%	58.23%	0.81
		64${}^{2}$ 128${}^{2}$ 256${}^{2}$	62.33%	74.25%	0.79
		16${}^{2}$ 64${}^{2}$ 128${}^{2}$	71.20%	69.23%	0.8
KAIST	RCNN	128${}^{2}$ 256${}^{2}$ 512${}^{2}$	25.25%	35.60%	0.4
		64${}^{2}$ 128${}^{2}$ 256${}^{2}$	20.36%	45.30%	0.2
		16${}^{2}$ 64${}^{2}$ 128${}^{2}$	29.20%	42.10%	0.3
	Faster RCNN-M	128${}^{2}$ 256${}^{2}$ 512${}^{2}$	27.20%	39.70%	0.32
		64${}^{2}$ 128${}^{2}$ 256${}^{2}$	32.20%	44.50%	0.28
		16${}^{2}$ 64${}^{2}$ 128${}^{2}$	37.50%	49.00%	0.49
	Faster RCNN	128${}^{2}$ 256${}^{2}$ 512${}^{2}$	29.90%	33.67%	0.5
		64${}^{2}$ 128${}^{2}$ 256${}^{2}$	18.50%	28.30%	0.4
		16${}^{2}$ 64${}^{2}$ 128${}^{2}$	18.90%	29.40%	0.19
	RPN+BF	128${}^{2}$ 256${}^{2}$ 512${}^{2}$	19.62%	34.02%	0.25
		64${}^{2}$ 128${}^{2}$ 256${}^{2}$	22.20%	30.12%	0.36
		16${}^{2}$ 64${}^{2}$ 128${}^{2}$	18.50%	27.60%	0.41
	SIFRCNN (Proposed)	128${}^{2}$ 256${}^{2}$ 512${}^{2}$	38.25%	60.33%	0.78
		64${}^{2}$ 128${}^{2}$ 256${}^{2}$	78.22%	60.58%	0.87
		16${}^{2}$ 64${}^{2}$ 128${}^{2}$	69.90%	70.09%	0.79

**Table 5:** Hardware specification for dataset validation and testing.

Hardware Specification for Validation	
System	Intel Core i9
RAM	16 GB
Graphics	NVIDIA RTX 2060
HDD	500 GB 7200 rpm

**Table 6:** Summary of testing and validation samples.

Process	Dataset	Time of Image Capturing	Total Images	Training Images	Testing Images	Total Pedestrian Detected
Origina training data	CityPerson	Daytime	14,523	10,166	4357	18,233
	Caltech	Daytime	15,840	11,088	4752	26,290
	KAIST	Nighttime	976	683	293	2166
Augmented data	CityPerson	Nighttime	12,266	8586	3680	1856
	Caltech	Nighttime	1584	1109	475	2629
	KAIST	Nighttime	828	580	248	1328
Validating data	CityPerson	Daytime	489	342	147	1877
	Caltech	Nighttime	300	210	90	62
	KAIST	Nighttime	797	558	239	399

## Data Availability

The data will be available on request.
